# The Effects of Warming and Nitrogen Addition on Soil Nitrogen Cycling in a Temperate Grassland, Northeastern China

**DOI:** 10.1371/journal.pone.0027645

**Published:** 2011-11-11

**Authors:** Lin-Na Ma, Xiao-Tao Lü, Yang Liu, Ji-Xun Guo, Nan-Yi Zhang, Jian-Qin Yang, Ren-Zhong Wang

**Affiliations:** 1 State Key Laboratory of Vegetation and Environmental Change, Institute of Botany, The Chinese Academy of Sciences, Xiangshan, Beijing, China; 2 Key Laboratory of Vegetation Ecology, Ministry of Education, Northeast Normal University, Changchun, Jilin Province, China; 3 Graduate University of Chinese Academy of Sciences, Yuquanlu, Beijing, China; 4 State Key Laboratory of Forest and Soil Ecology, Institute of Applied Ecology, Chinese Academy of Sciences, Shenyang, China; DOE Pacific Northwest National Laboratory, United States of America

## Abstract

**Background:**

Both climate warming and atmospheric nitrogen (N) deposition are predicted to affect soil N cycling in terrestrial biomes over the next century. However, the interactive effects of warming and N deposition on soil N mineralization in temperate grasslands are poorly understood.

**Methodology/Principal Findings:**

A field manipulation experiment was conducted to examine the effects of warming and N addition on soil N cycling in a temperate grassland of northeastern China from 2007 to 2009. Soil samples were incubated at a constant temperature and moisture, from samples collected in the field. The results showed that both warming and N addition significantly stimulated soil net N mineralization rate and net nitrification rate. Combined warming and N addition caused an interactive effect on N mineralization, which could be explained by the relative shift of soil microbial community structure because of fungal biomass increase and strong plant uptake of added N due to warming. Irrespective of strong intra- and inter-annual variations in soil N mineralization, the responses of N mineralization to warming and N addition did not change during the three growing seasons, suggesting independence of warming and N responses of N mineralization from precipitation variations in the temperate grassland.

**Conclusions/Significance:**

Interactions between climate warming and N deposition on soil N cycling were significant. These findings will improve our understanding on the response of soil N cycling to the simultaneous climate change drivers in temperate grassland ecosystem.

## Introduction

Anthropogenic increases in global atmospheric CO_2_ concentration are contributing to rising global temperatures over some areas, including northeastern China [1], [2]. Global anthropogenic N fixation now exceeds all natural sources of N fixation, and its products include greenhouse gases such as N_2_O that further contribute to climate change [3]–[5]. Many temperate ecosystems are predicted to experience rates of atmospheric N deposition as high as 2–5 g m^−2^ above preindustrial rates over this century [6]. Responses of soil carbon (C) storage to ongoing climatic and atmospheric changes (e.g. warming, N deposition) determine whether terrestrial ecosystems will act as a source or sink of the atmospheric CO_2_. Soil N processes are tightly coupled to the soil carbon cycle, while the soil N cycle may be a key process influencing ecosystem response to warming [7]. Moreover, as a main limiting factor for plant growth and net primary productivity [8], [9], understanding soil N availability and its response to climatic and atmospheric changes are vital for soil N dynamics and net primary production and global C budgets in terrestrial ecosystems [10], [11].

Previous studies involving field measurements and short- and long-term models have demonstrated that both warming and N addition individually alter soil N mineralization. Soil N mineralization has been found to increase with temperature under laboratory incubations through increasing soil microbial activity [12], [13]. Consequently, elevated temperatures applied with different techniques in the field are reported to stimulate net N mineralization rates in various biomes across the world [14]–[16]. In N-limited ecosystems, N deposition (or addition) has potential to stimulate soil N mineralization through direct fertilization which may increase microbial activity temporarily and by indirectly altering soil organic matter quality (e.g., lower soil C: N ratio) [17]–[20].

Concurrent changes in warming and N deposition may potentially trigger complex interactive influences on ecosystem functioning. Although several related studies have documented the combined effects of warming and N deposition on soil N mineralization and N storage in forest and temperate old field ecosystems [21], [22], mechanistic understanding of the interactive effect of warming and N deposition on soil N mineralization is still limited. Available results show that soil N availability and N leaching in high-elevation spruce and fir forests will increase in response to regional warming under N saturation condition [21]. Recently, Turner and Henry [22] reported that N addition did not increase extractable inorganic N in warmed plots compared to unwarmed plots in old field ecosystem, because added N was mostly captured by live plant roots and microbes or lost to atmosphere and suggest that warming over winter may amplify soil N losses under conditions of N saturation. Compared with forest and old field ecosystems, less is known about the interactive effects of warming and N deposition on soil N mineralization in temperate grassland ecosystems.

The temperate grassland in northeastern China is an important repository of carbon, and is sensitive to climate changes [1]. Both water and N are important limiting factors in this area [23]. To examine the responses of soil N mineralization to climate warming and increased N deposition, we conducted a manipulative experiment including increased topsoil temperature of approximately 1.8°C and N addition (10 g m^−2^ yr^−1^) in the grassland. We hypothesized that (1) warming and N addition would significantly increase potential soil N mineralization in this study site, (2) there would be additive effects of combined warming and N addition on potential soil N mineralization, because warming and N addition both can increase soil microbial activity. In addition, the observations in this study were from three precipitation contrasting growing seasons. Thus, we also test the impacts of precipitation variations on responses of soil N mineralization and N availability to warming and N addition.

## Results

### Soil microclimate

**Figure 1:**
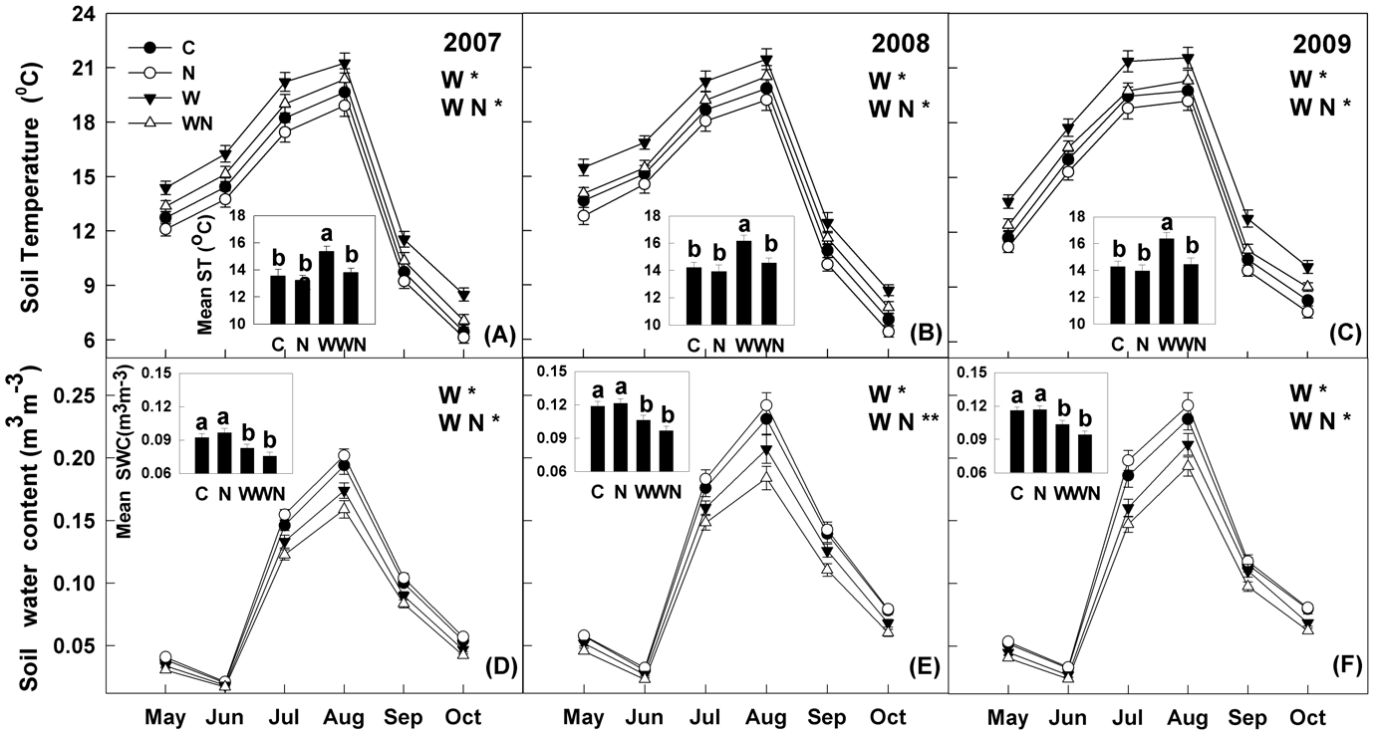
Seasonal variations of soil temperature (ST) and soil water content (SWC) at 15 cm depth in response to warming (1.8°C) and N addition (10 g m^−2^ yr^−1^) in the temperate grassland of northeastern China. Values show the means of ST and SWC from May to October in three years. The small pictures show the seasonal mean ST and SWC values of the three growing seasons under the four treatments. Vertical bars indicate standard errors of means (n = 6). Difference lowercase letters indicate statistically significant differences (*P*<0.05). C = control, N =  N addition, W =  warming, WN =  combined warming and N addition. *, ** represent significant at *P*<0.05 and *P*<0.01.

Seasonal fluctuations of both soil temperature and water content produced one-peak patterns, higher in summer and lower in spring and autumn in the three growing seasons (Fig. 1A–F). Warming significantly increased soil temperature at the 15 cm depth (Repeated measures ANOVAs, *F*_1, 20_ = 6.33, *P*<0.05, Fig. 1A–C), whereas N addition did not significantly impact soil temperature. There was a significant interaction between warming and N addition in affecting soil temperature (*F*_1, 20_ = 4.97, *P*<0.05), in that warming significantly increased soil temperature under ambient N level but not under N-enriched conditions. Soil water content (0–15 cm) was noticeably drier in 2007 than in 2008 and 2009 (*P*<0.001, Fig. 1D–F). Warming significantly reduced soil water content at the depth of 0–10 cm (*F*_1, 20_ = 6.74, *P*<0.05), whereas N addition showed no effect. Warming and N addition significantly interacted to affect soil water content (*F*_1, 20_ = 5.56, *P*<0.05); N addition showed a minor effect on soil water content under unwarmed plots but significantly decreased it under warmed plots.

### Treatment effects on control factors over soil N mineralization

**Figure 2:**
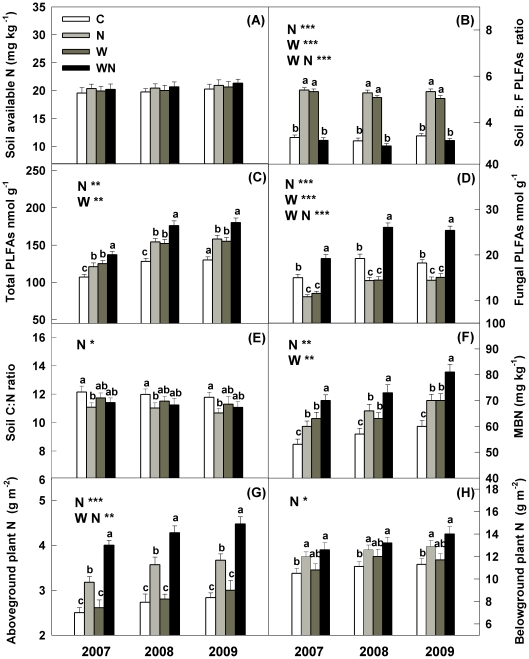
Responses of soil available N, soil bacterial to fungal PLFAs ratio (B: F), soil total PLFAs, fungal PLFAs, soil C: N ratio, soil microbial biomass N (MBN), aboveground plant N and belowground plant N to warming (1.8°C) and N addition (10 g m^−2^ yr^−1^) in the temperate grassland of northeastern China. Values show the seasonal means of soil B: F, total PLFAs, fungal PLFAs, C: N and MBN from 2007 to 2009. Vertical bars indicate standard errors of means (n = 6). Difference lowercase letters indicate statistically significant differences (*P*<0.05). *, ** and *** represent significant at *P*<0.05, *P*<0.01 and *P*<0.001. See Fig.1 for abbreviations.

Nitrogen addition increased soil bacterial to fungal PLFAs ratio (B: F) by 60%, 63% and 56% (*P*<0.001, Fig. 2B), total PLFAs by 13.2%, 20.3% and 21.5% (*P*<0.01, Fig. 2C), soil microbial biomass N (MBN) by 13.2%, 15.7% and 16.6% (*P*<0.01, Fig. 2F), aboveground plant N content by 27%, 30.4% and 29.4% (*P*<0.001, Fig. 2G) and belowground plant N content by 14.2%, 13.5% and 11.2% (*P*<0.05, Fig. 2H) in 2007, 2008 and 2009, respectively. However, N addition decreased fungal PLFAs by 28%, 25.4% and 21% (*P*<0.001, Fig. 2D), and soil C: N by 10.2%, 9.7% and 10% (*P*<0.05, Fig. 2E) in 2007, 2008, and 2009, respectively.

Warming increased soil B: F by 58%, 57% and 47% (*P*<0.001, Fig. 2B), total PLFAs by 16.8%, 18.7% and 19.2% (*P*<0.01, Fig. 2C), and MBN by 17.7%, 12.5% and 16.6% (*P*<0.01, Fig. 2F) in 2007, 2008 and 2009, respectively. However, warming decreased fungal PLFAs by 23.2%, 24.7% and 17.4% (*P*<0.001, Fig. 2D) in 2007, 2008, and 2009, respectively.

There were significant interactions between warming and N addition on soil B: F (*P*<0.001), soil fungal PLFAs (*P*<0.001), and aboveground plant N content (*P*<0.01) during the three growing seasons, as it was found that the increases in B: F and fungal PLFAs were significantly smaller and higher than would be expected if the two factors acted additively (Fig. 2B, D). Moreover, warming showed no effect on aboveground plant N content but showed positive effects under enriched N condition (Fig. 2G).

### Control factors over soil N mineralization at the temporal and spatial scales

**Figure 3:**
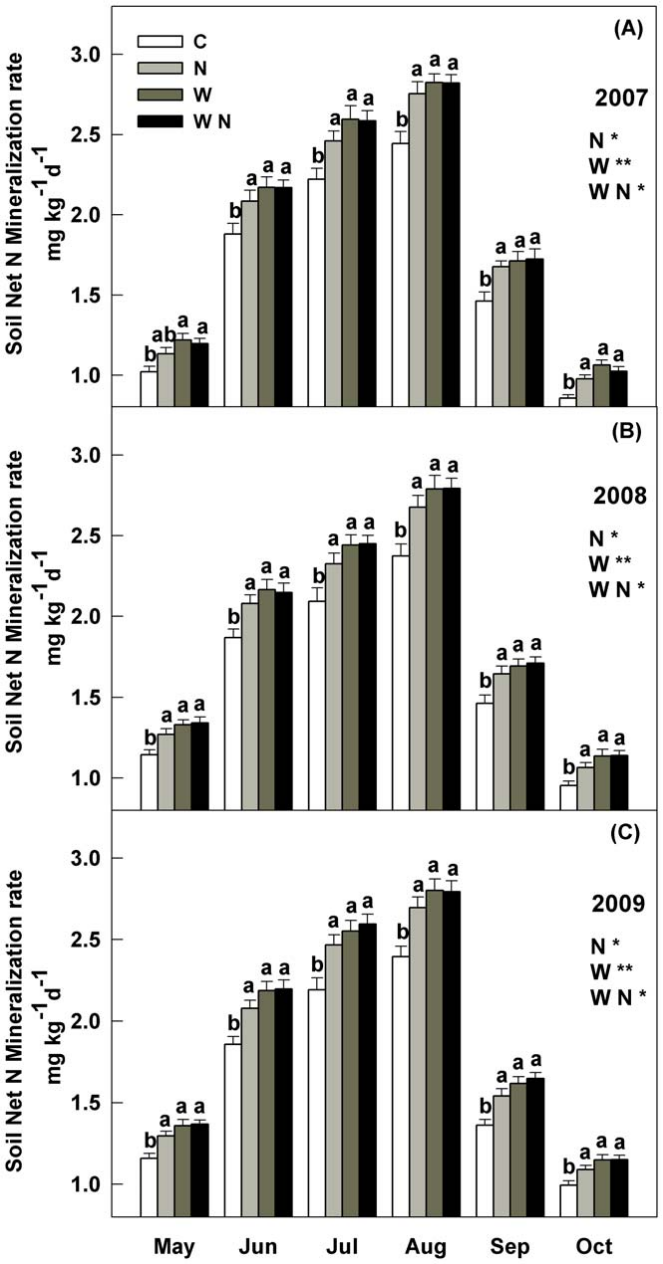
Seasonal dynamics of soil net N mineralization rate (NMR) at 15 cm depth under warming (1.8°C) and N addition (10 g m^−2^ yr^−1^) treatments in temperate grassland of northeastern China. Values show the monthly means of soil NMR from May to October in three years. Vertical bars indicate standard errors of means (n = 6). Difference lowercase letters indicate statistically significant differences (*P*<0.05). *, ** represent significant at *P*<0.05 and *P*<0.01. See Figure 1 for abbreviations.

In general, the seasonal dynamics of soil net N mineralization rate (NMR) and net nitrification rate (NNR) showed unimodal pattern with the higher values in summer and lower values in spring and autumn over the three growing seasons (Fig. 3A–C, 4A–C). Soil NMR and NNR across four treatments in 2007 were significant lower than those in 2008 and 2009 (*P*<0.001). Across the three growing seasons, stepwise multiple regression analysis of soil NMR (or NNR) with control factors indicated the combination of soil temperature (partial R^2^ = 0.43 in NMR, *P*<0.001; partial R^2^ = 0.42 in NNR, *P*<0.001) and soil water content (partial R^2^ = 0.29 in NMR, *P*<0.01; partial R^2^ = 0.32 in NNR, *P*<0.01) explained 72% and 74% of the seasonal variations of soil NMR and NNR. These results suggest that all the concurrent seasonal variations of soil temperature and soil water content contributed to the temporal fluctuations of soil N mineralization.

**Figure 4:**
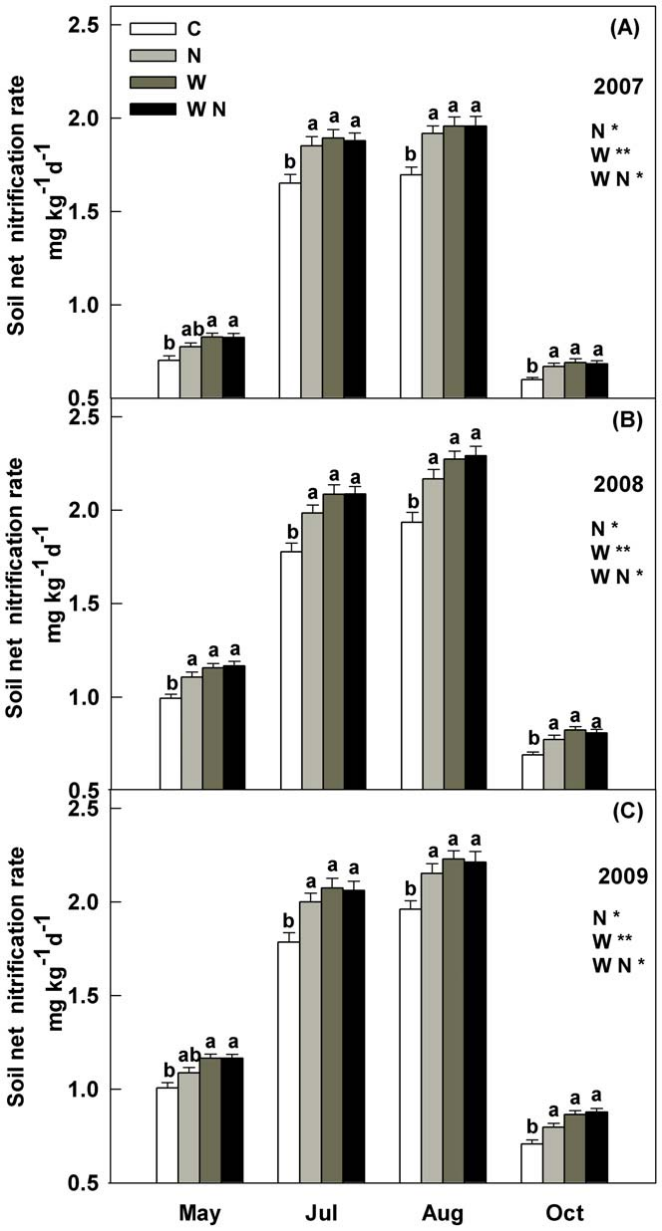
Seasonal responses of soil net N nitrification rate (NNR) at 15 cm depth to warming (1.8°C) and N addition (10 g m^−2^ yr^−1^) in temperate grassland of northeastern China. Values show the monthly means of soil NNR from May to October during the three growing seasons. Vertical bars indicate standard errors of means (n = 6). Difference lowercase letters indicate statistically significant differences (*P*<0.05). *, ** represent significant at *P*<0.05 and *P*<0.01. See Figure 1 for abbreviations.

Across the 24 plots, stepwise multiple regression analyses demonstrated that soil NMR showed a positive linear correlations with mean soil total PLFAs and soil B: F, and negative linear correlation with soil C: N. Fifty-eight percent of the spatial variation in soil NMR could be explained by the soil B: F (partial R^2^ = 0.24, *P*<0.01) and total PLFAs (partial R^2^ = 0.34, *P*<0.01) in 2007. In 2008, soil B: F (partial R^2^ = 0.27, *P*<0.01), total PLFAs (partial R^2^ = 0.38, *P*<0.01) and soil C: N (partial R^2^ = 0.8, *P*<0.01) together accounted for 73% in NMR. In 2009, 71% of the spatial variability in NMR could be attributable to the combination of soil B: F (partial R^2^ = 0.25, *P*<0.01), total PLFAs (partial R^2^ = 0.39, *P*<0.01) and soil C: N (partial R^2^ = 0.7, *P*<0.01).

Similar to soil NMR, stepwise multiple regression analyses showed that soil B: F (partial R^2^ = 0.26, *P*<0.01) and total PLFAs (partial R^2^ = 0.32, *P*<0.01) contributed to 58% of the spatial variation in NNR in 2007. In 2008, B: F (partial R^2^ = 0.24, *P*<0.01) and total PLFAs (partial R^2^ = 0.35, *P*<0.01) were responsible for 59% of the spatial variation in NNR. In 2009, 67% of the changes in soil NNR can be explained by the combination of soil B: F (partial R^2^ = 0.23, *P*<0.01), total PLFAs (partial R^2^ = 0.36, *P*<0.01) and soil C: N (partial R^2^ = 0.8, *P*<0.01). These results suggest that abiotic (soil C: N) and biotic (soil microbial community structure and microbial biomass) factors played important roles in regulating spatial variations in soil N mineralization in the temperate grassland.

### Main effects on soil N mineralization

During the three years, the main effects of N addition and warming significantly increased soil NMR and NNR, and year did not interact with warming (or N addition) to affect NMR and NNR. Warming treatments had significantly greater soil NMR and NNR than the control, resulting in 18%, 16.9% and 17% higher soil NMR, and 14.8%, 17.6% and 18.3% higher NNR in 2007, 2008, and 2009, respectively (*P*<0.01, Fig. 3A–C, 4A–C). N addition enhanced soil NMR by 12.3%, 11.8% and 11.9%, and NNR by 11%, 12.9%, and 15.3% in 2007, 2008, and 2009, respectively (*P*<0.05, Fig. 3 A–C, 4 A–C).

### Interactive effect on soil N mineralization

Significant interactions between warming and N addition on soil NMR and NNR were observed (*P*<0.05, Fig. 3 A–C, 4 A–C). Soil NMR in the combined warming and N addition treatments was only 17.5–20.1% (*P*<0.01) higher than in the control plots in all three years, while the additive effects of individual warming and N addition would much greater (28.7–30.3%). The increases in NNR (16.2–20.2% in three years) in the combined factors treatments were significant lower than the summed effects of warming and N addition separately (28.6–31.6%).

## Discussion

### Response of soil N mineralization to warming

In line with hypothesis 1, warming (+1.8°C) had positive effects on potential net N mineralization and net nitrification rates, as expected. The rapid increase in soil potential net N mineralization was possibly due to more soil microbial biomass (total PLFAs) (Fig. 2C) and active microbial activity in the warmed soil, and consequently a significant increase in soil organic matter decomposition. This is supported by observations in other grasslands [14], [16]. However, soil available N concentration (AN) was not increased in the warmed plots (Fig. 2A) after the three growing seasons. This may be primarily attributed to the immobilization of soil microbes (Fig. 2F) and loss through emissions of greenhouse gases (e.g. NO, N_2_O, N_2_) from denitrification because of less N uptake by plant (Fig. 2G, H). The rapid responses of soil N mineralization to increases in temperature observed in this study indicate that the temperate grassland soil N mineralization is sensitive to global warming.

### Response of soil N mineralization to N addition

In N-limited ecosystems, N deposition has widely been shown to stimulate soil N mineralization [18]–[20]. Similar results have been found in this study. The most probable explanation for the positive N effect is that N enrichment may increase microbial activity temporarily, either directly after fertilization causing a priming of soil organic matter mineralization [24], or indirectly when large quantities of lower C: N ratio residues are incorporated into soil organic matter, reducing soil C: N ratio (Fig. 2E) and then increasing N release during decomposition [17], [25]. This is in keeping with the observations in temperate grassland in Northern China [26] and in an acidic and calcareous grassland in Britain [27]. However, N addition had a minor effect on soil AN concentrations (Fig. 2A) after the three growing seasons. This is likely due to the uptake of soil AN by plants and soil microbes, which then contributes to the significant increase of aboveground and belowground plant N content and MBN (Fig. 2F, G, H). These results therefore suggest that the increasing anthropogenic N deposition may be partly directly utilized by plants and microbes in the temperate grassland.

### Interactive effects of combined warming and N addition

Given that warming and N addition can significantly increase soil N mineralization, it is reasonable to assume there would be additive effects of these two treatments on soil N mineralization. However, in contrast to our expectation, the increases in soil N mineralization were significantly smaller than would be expected if the two factors acted additively.

The combined warming and N addition caused an antagonistic effect. This antagonistic effect could be largely explained by the relative changes of soil microbial community structure (B: F) and aboveground plant N content in the combined warming and N addition treatment. The relative lower B: F (Fig. 2B) due to the increase of fungal biomass (Fig. 2D) under combined factors than the single factors may reduce the decomposition process. Firstly, fungal hyphae have long been recognized to enmesh microaggregates (<250 µm) into macroaggregates (>250 µm) [28], [29], which contributes to SOM stabilization and protection by enhancing soil aggregation. Secondly, fungal cell walls contain more polymers such as melanin and chitin, which can persist in soil for years and account for significant pool of SOM compared with bacteria [30]. In addition, the strongly positive response of aboveground plant N content to combined warming and N addition (Fig. 2G) may also lead to more N uptake by local plants and the rapid decline of soil AN content, which could suppress the positive N effects on soil N mineralization. Similar to warming or N addition, combined warming and N addition only slightly increased soil AN concentration after the three growing season which might have been caused by absorption by local plants and microbes (Fig. 2F, G, H). Thus, these findings suggest that the multifactor effects can differ from simple combinations of single-factor responses. Considering the unprecedented climate warming associated with increasing N deposition under global climate change, multifactor experiments are needed in the future to fully understand the impacts of climatic and atmospheric changes on ecosystem N cycling.

### Impact of precipitation variation

In this study, soil water content partly controlled the temporal fluctuations of soil N mineralization indicating that soil water content is an important factor for soil N mineralization. In addition, lower seasonal mean soil NMR and NNR in the dry year (2007) than in wetter years (2008 and 2009) in all treatments (*P*<0.001) suggest that critical roles of soil water availability in regulating soil N mineralization in the temperate grassland in northeastern China. This is supported by some previous observations [31], [32]. However, similar positive responses of soil N mineralization to warming (or N addition) amongst the three growing seasons imply that the responses of soil N mineralization to warming and N deposition were independent of precipitation variations in the temperate grassland. Thus, irrespective of the dependence of intra- and inter-annual variations in soil N mineralization on precipitation, precipitation variability may not alter the impacts of warming and N addition on soil N mineralization in the temperate grassland in northeastern China.

### Conclusions

Both warming and N addition enhanced soil N mineralization in the three consecutive years with fluctuating precipitation in the temperate grassland. Contrary to our prediction, combined warming and N addition produced a significantly interactive effect on soil N mineralization. This result might be ascribed to the relative changes in soil microbial community structure and plant N content. These findings highlight the importance of changes in abiotic (soil temperature, soil water content and soil C: N) and biotic (plant N content, soil microbial community structure and microbial biomass) in regulating ecosystem function in the temperate grassland. Moreover, although the drier year (2007) contributed to lower soil N mineralization, it did not alter the impacts of warming and N addition on soil N mineralization. Extra available N addition would be partly taken up by plants or soil microbes. The observations in the present study will facilitate long-term projections of regional soil N cycling and improve our understanding of soil N cycling response to the simultaneous climate change drivers in temperate grassland ecosystems.

## Materials and Methods

### Ethics Statement

The Research Station of Songnen Grassland Ecosystem (44°45'N, 123°45'E) is a department of Northeast Normal University. This study was approved by Key Laboratory of Vegetation Ecology, Ministry of Education, Northeast Normal University and State Key Laboratory of Vegetation and Environmental Change, Institute of Botany, the Chinese Academy of Sciences.

### Study site and Experimental design

The experiment was conducted at the Research Station of Songnen Grassland Ecosystem (44°45'N, 123°45'E), Jilin Province, northeastern China from 2006. The grassland is situated at the eastern edge of the Eurasian steppe and is characterized as Eurasian continental temperate grassland. Mean annual precipitation (1980–2006) was approximately 410 mm with 90% from May to October, while the total precipitation in 2007 (275.9 mm), 2008 (384.2 mm) and 2009 (390 mm) was 32.7%, 6.2% and 4.9% lower than the mean annual precipitation. Annual average air temperature was 4.9°C, and annual average land surface temperature was 6.2°C. Most of the grassland had a sodic saline meadow soil. Soil pH was 8.2, with 3% to 4% organic matter in the surface layer. The vegetation at the site was dominated by the perennial grass *Leymus chinensis* (Trin.) Tzvel., whereas *Kalimeris integrifolia* Turcz. Ex DC., *Carex duriuscula* C. A. Mey., and *Rhizoma Phragmitis* were also present at lower density. Shoot heights were typically 30–40 cm, and the maximum litter accumulation about 0.5 kg m^–2^ [33].

The experiment was employed in a complete randomized block factorial experimental design with warming and N addition as fixed factors and two levels for each factor. There were four treatments (control, warming, N addition and combination of warming and N addition), with six replicates for each treatment. The size of each plot was 2 m×3 m. All the warmed plots were heated continuously by infrared radiators (Kalglo Electronics Inc. Bethlehem, PA, MSR-2420, USA) suspended 2.25 m over the plot center. In each control or N addition plot, one ‘dummy’ heater with the same shape and size as the shading effects of the infrared radiator. All the heaters under the warming treatments were set at a radiation output of approximately 1700 W. He et al. estimated that airborne N up to 80–90 g m^−2^ yr^−1^ and higher N deposition would occur in the future owing to land-use change and anthropogenic activities [34], [35]. In addition, Bai et al. estimated that the community saturation of N deposition rates was approximately 10.5 g m^−2^ yr^−1^ in this temperate grassland ecosystem [36], even though the atmospheric N deposition in this temperate ecosystem was as high as 2.7 g m^−2^ yr^−1^ in recent ten years [35]. Thus, in the fertilized plots, we added a pulse of aqueous ammonium nitrate on the first day in May at a rate of 10 g m^−2^ yr^−1^. Ambient N plots received the same amount of water as the N addition plots in the spring (equivalent to<2 mm of rain), but no added N.

### Soil microclimate measurements and samplings

Soil temperature and water content were measured by using an ECH_2_O dielectric aquameter (Em50, USA), which automatically measured soil temperature and soil water content at a depth of 15 cm at 8: 00–9: 00 in late May, mid-June, mid-July, early August, mid-September and mid-October in 2007, 2008 and 2009. Five measures were taken and averages of the five measures were stored as the mean value per plot.

Soil core samples were collected concurrently from two random locations in each plot for determination of soil potential net N mineralization and nutrient. Soil samples were taken with a cylindrical soil sampler (5 cm inner diameter, 15 cm length) for the 0–15 cm layer, and then immediately preserved at 4°C in a cooler for transport to the laboratory. The fresh samples were processed using a 2 mm sieve and manually cleaned of any visible plant tissues. The two replicates from the same depth on each plot were pooled and treated as a single sample for analysis.

### Soil N mineralization and nutrient measurements

Soil N mineralization was assessed in the lab using an aerobic procedure [37], [38]. In order to minimize disturbance of microbial activity, fresh (not dried) soil samples were used [39]. A soil subsample (about 10 g dry weight) was mixed thoroughly with 10 g acid-washed sand (The added acid-washed sand can widen the soil pore and make for thorough soil N mineralization) and transferred to a 250 ml triangle bottle, then was adjusted soil water content to 60% water-holding capacity. The bottles were covered with 0.01 mm thick plastic film and were incubated at 25°C for fourteen days in a constant temperature cabinet (SHP-25, China). During incubation, the bottles weighed periodically, and had deionized water added to maintain a constant water content. To analyze the available N (NH_4_^+^-N + NO_3_^-^-N) concentrations, each un-incubated and incubated soil sample was added 100 ml of 2 M KCl solution. The mixture of soil and extractant was shaken for 1 hour on a reciprocal shaker, and then centrifuged for 10 minutes. Supernatant was collected with a pipette and was frozen for later. Soil available N (NH_4_^+^-N + NO_3_^-^ -N) concentrations in the incubated and un-incubated soil subsamples were estimated by steam distillation of the supernatants with subsequent titration [40]. The NO_3_^-^ -N concentrations were determined by UV - photometrically at 210 nm [41]. Soil net N mineralization rate and net nitrification rate were calculated as the changes in available N (NH_4_^+^-N + NO_3_^-^-N) and NO_3_^-^-N in the initial and incubated subsamples.

Soil microbial biomass N (MBN) was measured using a chloroform fumigation-direct extraction procedure [42]. MBN were calculated as the difference in available N contents between the fumigated and the unfumigated samples using conversion factors (k_en_) of 0.45 [43]. Soil total N (TN) was determined via digestion with H_2_SO_4_ by the Kjeldahl method [44]. Soil organic C (SOC) was measured with the dichromate oxidation method [45].

### Microbial community structure

The PLFAs were extracted and quantified from 8.0 g (dry weight equivalent) soils using a procedure previously described by Bossio et al. [46]. The separation and identification of extracted PLFAs were carried out according to the standard protocol of the Sherlock Microbial Identification System V3.1 (MIDI, 1999) and a Gas Chromatograph (Hewlett Packard 6850, USA). Fatty acid nomenclature used in the present research was as that defined by Bossio et al. [46]. The following fatty acids i15: 0, a15: 0, i16: 1c, i16: 0, 16: 1w7c, i17: 0, 17: 1w6c, a17: 0, 17: 0cy, 18: 1w7c, 18: 1w5c and 19: 0cy were chosen to represent the PLFAs of the bacterial group [47], [48]. Also three fatty acids (16: 1w5c, 18: 2w6, 9c and 18: 1w9c) were used to represent the fungal group [49]. The ratio of bacterial and fungal fatty acids was also included in the data analysis. This ratio has often been used as the indicator of the change in the soil microbial community structure [50].

### Plant N content measurements

Twenty replicate plant samples in each plot with intact root systems were collected for fall 2007, 2008 and 2009. We used *L. chinensis* as indicator species because it was abundant and uniformly distributed across the study site. Each plant was divided into above- and belowground parts, oven-dried at + 70°C. Each individual above- and belowground sample was analyzed for plant aboveground and belowground N contents by the Kjeldahl method after acid digestion [44].

### Statistical Analysis

Seasonal mean values used in this study were calculated from the monthly mean values, which were first averaged from all measurements in the same month. Repeated measures ANOVAs were used to examine the temporal (inter- or intra-annual) variations and the effects of warming and N addition on soil N mineralization, soil temperature, soil water content, soil total PLFAs, fungal PLFAs, B: F, MBN, AN, C: N, and above- and belowground plant N content. Between-subject effects were evaluated as warming, N addition, and their interactions, and within-subject effects were year (or measuring times within season) and its interactions with warming or N addition. Stepwise multiple linear analyses were used to determine the relationships of soil NMR (or soil NNR) with control factors. Statistical analyses were conducted using SPSS (SPSS 11.0 for windows, USA).
